# Incipient Insanity and Home Life

**Published:** 1921-06-25

**Authors:** 


### INCIPIENT INSANITY AND HOME LIFE.

**By a SOCIAL WORKER.**

Few of the sorrows and anxieties of everyday life are so carefully veiled as those of families, most of all, in many piteous cases, of the mother of a family which contains a certified or "borderland" case. The poor lady—I think now of those who are "in Society" in their town or neighbourhood—scarcely dares to speak of her fears to others; distressing symptoms she broods over alone, knowing that eccentricity or unseemly conduct in her growing son or daughter will be attributed to her own mismanagement by outsiders, perhaps even more severely so by relations. She tries coaxing and finds herself accused of "spoiling," she adopts some severity and incurs the resentment of the difficult boy or girl and the censure of the onlookers to whom, probably, impassioned complaints are made. The family doctor is not very helpful; perhaps he shrugs his shoulders and suggests that "the back of a hairbrush" was resorted to in his youth with wholesome results in such cases. If things assume a more serious aspect, especially in the case of a girl, he murmurs something about *dementia præcox*, and recommends a specialist.

At an informal conference lately called by Lady Selborne, as President of the National Council of Women, where two women specialists urged the need for better training of doctors in mental symptoms and their treatment, it was resolved that efforts should be made towards this end. Much danger might be averted if the family physician were better qualified to see where unstable conditions might rightly be ascribed to hysteria due to overwork, to difficult development, or some ailment needing treatment, and where a case should promptly be referred to examination by an alienist.

At present, here is the point where the dreaded stigma begins. The mere fact that a son or daughter has been examined by a brain specialist will, if possible, be kept secret. Marriages for other daughters may, the mother knows, be prevented because a rumour is abroad that there is insanity in the family. It is a poor consolation to be told that genius is more common in such families, the fact remains that the stigma is there. If the patient recovers at once, it may be lived down, but recovery may be hindered in the case of a sensitive young person who has heard that whisper. Borderland cases are often hypersensitive, frequently vain, with often a painful mixture of want of self-confidence and desperate self-assertion. Depression constantly falls upon the whole nature like a chill shadow, and to a young girl one of the most depressing circumstances of life is a feeling that she is unlike others. Should the verdict of the specialist be that a time of being under observation in a doctor's house is necessary, or a trip in charge of a trained nurse desirable, this impression may be deepened. The doctor has perhaps one or two patients who are certified, and, even if these are kept apart, some knowledge of their condition is unavoidably gained. Or there may be others under observation whose eccentricities are evident. The idea of insanity is presented to the young mind, and is sure to obtrude itself; even if a nurse out of uniform is the companion on a trip, the fact that she is a care-taker cannot be blinked entirely.

If, on the other hand, the patient is allowed to be at home, the anxiety may be very great. As in all cases of illness, the families most hard hit by such circumstances are those of the poorer gentry and professional people, who cannot give special luxuries to one member of their party, or provide the peculiar care that may be needed. The daughter at home who is a borderland case may do infinite mischief, to younger sisters especially, by infusing into their minds her own diseased ideas. Outside the house, by irresponsible behaviour of all kinds, perhaps very carefully concealed from the parents, she may bring the family into grave disrepute and herself into major or minor scrapes which harm herself and them. She may at times be in a condition in which she would be better certified for her own sake and theirs; yet they will avoid this if possible because of the "disgrace," and because, also, they fear that if the girl were thus confined her future would be ruined. To realise her condition might, they feel, permanently impair her reason; while if she regained her liberty by showing herself sensible when interviewed by the Commissioners she might well return full of anger and resentment towards the relations who had tried to seclude her. It would be hard to convince her that affection could prompt such action. In the case of a son such a position of things is apt to result in his flinging himself out of all relations with his own people and going overseas, to be heard of at intervals or not for many years, an unhappy wanderer whom the family subsidise rather vainly and speak of but little.

Among the poorer classes this grief is lessened by the fact that the dread stigma of family insanity does not trouble them so much. Their suffering is rather from the male borderland case, of whom they go in fear when he has had drink, and who is at most times a drain on the family resources from never sticking to work, or from constant dismissals on account of violent or irrational behaviour. And in the female case immorality, dishonesty, or unclean habits may be grievous trials to respectable people. "She had ought to be put away, but there—we don't want to be hard on her," may be the verdict.

Will it be possible, by systems of voluntary treatment in a new type of home or of hospital ward, to abolish the absurd sense of ignominy that still attaches to brain troubles? That is after all the great hindrance, and the fact that crowds of shell-shocked men have been treated, and that the words "mental hospital" is tending to replace "asylum" in our language, should help. The further—very serious—question is, How, if cases are to be encouraged to come willingly for examination and treatment, are we to deal with those who should, for their own sake and that of others, be kept in confinement? As to the painful position of those recovered cases who are unwilling to return to family life because of the shadow which they feel to rest upon them, the saner point of view which public opinion should attain must have an excellent and most cheering effect. Let us hope for a sound measure before long.

